# RhoA/ROCK-YAP/TAZ Axis Regulates the Fibrotic Activity in Dexamethasone-Treated Human Trabecular Meshwork Cells

**DOI:** 10.3389/fmolb.2021.728932

**Published:** 2021-09-06

**Authors:** Zhicheng Liu, Shanshan Li, Xiuqing Qian, Lin Li, Haixia Zhang, Zhicheng Liu

**Affiliations:** ^1^ School of Biomedical Engineering, Capital Medical University, Beijing, China; ^2^ Beijing Key Laboratory of Fundamental Research on Biomechanics in Clinical Application, Capital Medical University, Beijing, China

**Keywords:** dexamethasone, RhoA/ROCK, YAP/TAZ, fibrotic activity, human trabecular meshwork cells

## Abstract

High intraocular pressure (IOP) is a major risk factor for glaucoma, a leading cause of irreversible blindness. Abnormal fibrotic activity in the human trabecular meshwork (HTM) cells is considered to be partly responsible for the increased resistance of aqueous humor outflow and IOP. This study aimed to identify the fibrotic pathways using integrated bioinformatics and further elucidate their mechanism of regulating fibrotic activity in dexamethasone (DEX)-treated HTM cells. Microarray datasets from the GEO database were obtained and analyzed by GEO2R. Bioinformatics analyses, including GO and KEGG analyses, were performed to explore biological functions and signaling pathways of differentially expressed genes (DEGs). The fibrotic pathways and targets were determined by western blot, RT-qPCR, or immunofluorescence staining. The cellular elastic modulus was measured using an atomic force microscope. A total of 204 DEGs, partly enriched in fibrotic activity (collagen-containing ECM, fibroblast activation) and Rap1, Ras, TGF-β, and Hippo pathways, were identified. Experimental results showed that DEX induced fibrotic activity and regulated the expression of RhoA/ROCK in HTM cells. Similarly, the constitutively active RhoA (RhoAG14V) also promoted the fibrotic activity of HTM cells. Mechanistically, RhoAG14V induced the expression and nuclear translocation of YAP/TAZ to produce CTGF. Moreover, inhibition of ROCK or YAP decreased the expression of Collagen I and α-SMA proteins induced by DEX or RhoAG14V in HTM cells. In conclusion, these results indicate that RhoA/ROCK-YAP/TAZ axis plays a crucial role in regulating the fibrotic activity of DEX-treated HTM cells.

## Introduction

Glaucoma, characterized by optic disc damage and visual field loss, is the second leading cause of blindness globally (Quigley and Broman, 2006). High intraocular pressure (IOP) is known to be related to increased resistance to the outflow of aqueous humor (AH) of the eye (Tamm, 2009). Lowering IOP by reducing the resistance of AH outflow has become the most commonly used treatment method for glaucoma. Since the trabecular meshwork (TM) plays a key role in maintaining normal AH outflow and regulating IOP, TM tissue intervention is an important clinical treatment modality to lower IOP (Tamm, 2009; Stamer and Clark, 2017). Therefore, identification of the mechanism of targeting human trabecular meshwork (HTM) cells may help in developing effective strategies to treat glaucoma.

Glucocorticoids are used to treat various ocular inflammatory diseases involving a variety of intraocular tissues (Overby and Clark, 2015). However, prolonged treatment with glucocorticoids, such as dexamethasone (DEX), can cause glucocorticoid-induced ocular hypertension (Overby and Clark, 2015; Patel et al., 2017; Patel et al., 2019), possibly leading to glaucoma and permanent vision loss. The elevation of IOP has been reported to be associated with an increase in TM tissue stiffness, and DEX is an important factor for causing stiffness of the TM tissue (Wang et al., 2017). Moreover, DEX has been shown to cause morphological and biochemical changes in the TM tissue, such as extracellular matrix (ECM) accumulation Raghunathan et al. (2015), which is a typical characteristic of fibrosis. Thus, HTM cells treated with DEX are used as a model of fibrotic activity *in vitro*. Due to the development and application of high-throughput sequencing and bioinformatics tools, disease-related biomarkers have been effectively identified. Gene expression profiling on the online public databases has been used to explore differentially expressed genes (DEGs) and signaling pathways involved in the physical and pathological processes of diseases (Tu et al., 2018). In this study, system-wide profiling of DEX-treated HTM cell samples was explored to obtain biological processes and signaling pathways of DEGs using bioinformatics analyses.

Cell-matrix mechanoreceptors including integrin and cytoskeletal proteins play an important role in DEX-induced ECM remodeling (Yemanyi et al., 2020). RhoA acting as one of the cytoskeletal proteins regulates the dynamic assembly of cytoskeleton through activating Rho-associated coiled-coil containing protein kinase (ROCK) (Tan et al., 2018; Tan et al., 2019; Tan et al., 2020). ROCKs are the major downstream effectors of Rho GTPase and ROCK activation regulates the clustering of actin stress fiber in the cytoskeleton, which promotes actomyosin contractility and endogenous tension (Rao and Epstein, 2007; Tian et al., 2009). ROCK inhibitor has been shown to decrease cell tension and actin crosslinking. The yes-associated protein (YAP)/transcriptional coactivator with PDZ-binding motif (TAZ) that sense and transmit mechanical signals are essential transcriptional coactivators of the Hippo pathway (Meng et al., 2016). YAP/TAZ have been implicated as the nuclear relay of cytoskeletal changes (Reddy et al., 2013) mediated by stiffer substrates (Raghunathan et al., 2013; Thomasy et al., 2013), topography (Raghunathan et al., 2014), and cyclic stretch (Cui et al., 2015). In nuclear, YAP/TAZ binding with TEA domain family members (TEADs) regulate gene transcription of the cellular communication network family, such as connective tissue growth factor (CTGF), a matricellular protein participating in fibrosis (Lin et al., 2017). At present, the intrinsic relationship between RhoA/ROCK and YAP/TAZ and their role in regulating fibrotic activity remain unclear in DEX-treated HTM cells. Due to RhoA/ROCK being associated with intracellular tension, we hypothesized that RhoA/ROCK activation may promote the fibrotic activity of HTM cells via YAP/TAZ.

## Materials and Methods

### Microarray Data Acquisition and Bioinformatics Analyses

In this study, publicly accessible data were identified from the Gene Expression Omnibus (GEO) database to obtain microarray datasets. The datasets were searched from the GEO database with the keywords “dexamethasone” and “trabecular meshwork cells.” Four microarray datasets including GSE6298, GSE65240, GSE124114, and GSE16643 were identified. GSE6298 did not contain control samples and GSE65240 only included two control samples and two DEX-treated cell samples. Therefore, we selected GSE124114 and GSE16643 for subsequent analyses. In total, nine DEX-treated HTM cell samples and nine normal HTM cell samples were obtained in GSE124114 (Faralli et al., 2019), whereas six DEX-treated HTM cell samples and six normal HTM cell samples were obtained in GSE16643 (Nehme et al., 2009). Then, we used GEO2R to analyze microarray datasets and identify DEGs between the control group and the DEX-treated group. The threshold set for DEGs was a |fold change| ≥ 1.5 and *p* < 0.05. The common DEGs were selected with a Venn diagram. Ultimately, Gene Ontology (GO) and Kyoto Encyclopedia of Genes and Genomes (KEGG) enrichment analyses were conducted for DEGs by R “clusterProfiler,” “org.Hs.eg.db,” and “enrichplot” packages.

### Cell Culture and Treatment

HTM cells were purchased from ScienCell Research Laboratories. The cells were maintained in Dulbecco’s modified Eagle’s medium/Nutrient Mixture F-12 (DMEM/F12, 11330-032, Gibco) containing 10% fetal bovine serum (FBS, 10091-148, Gibco), 100 μg/ml streptomycin, and 100 U/ml penicillin solution (SV30010, Hyclone) and were cultured at 37°C with 5% CO_2_ in a humidified atmosphere. Then, the cells were treated with 1 μM DEX (HY-14648, MedChemExpress) for 72 h followed by 10 μM Y-27632 (ROCK inhibitor, HY-10071, MedChemExpress) for 24 h. Before treatment, HTM cells were starved for 24 h in serum-free DMEM/F12.

### Cell Transfection

The constitutively active RhoA (RhoAG14V) was provided by Beijing Syngentech Co., Ltd. The small interfering RNA of YAP (siYAP) were provided by Hanbio Biotechnology Co., Ltd. The siRNA sequences were as follows: small interfering of control (sictrl) sense, 5′-TTC​TCC​GAA​CGT​GTC​ACG​TAA-3′; siYAP sense, 5′-GGA​CTA​AGC​ATG​AGC​AGC​TAC​AGT​G-3′. The cells were transfected with lentivirus at 20 multiplicities of infection for 24 h, and the completed medium was replaced and further incubated for 48 h at 37°C. The transfection efficiency of lentivirus in HTM cells was evaluated using western blot.

### Western Blot

HTM cells were treated with RIPA buffer containing 1% protease and phosphatase inhibitor cocktail followed by centrifugation at 14,000 rpm for 15 min to extract proteins. Then, the supernatants were subjected to the bicinchoninic acid protein assay, and proteins were separated on polyacrylamide gels through electrophoresis. Subsequently, the proteins were transferred to PVDF membranes. The membranes were blocked in 5% skimmed milk for 1 h at room temperature and treated with specific primary antibodies overnight at 4°C. The primary antibodies were as follows: ROCK2 (1:10,000, ab125025, Abcam), Collagen I (1:1,000, ab260043, Abcam), α-Smooth muscle actin (α-SMA, 1:10,000, ab124964, Abcam), RhoA (1:1,000, 2,117, CST), YAP (1:1,000, 14,074, CST), YAP/TAZ (1:1,000, 8,418, CST), phosphorylated YAP (pYAP, 1:1,000, 13,008, CST), phosphorylated TAZ (pTAZ, 1:1,000, 59,971, CST), CTGF (1:1,000, 23936-1-AP, ProteinTech), and β-actin (1:2,000, 20536-1-AP, ProteinTech). The next day, the membranes were washed and incubated with the horseradish peroxidase-conjugated goat anti-rabbit IgG (1:10,000, 111-035-003, Jackson ImmunoResearch Laboratories) at room temperature for 1 h. The membranes were visualized using a chemiluminescence imager. The band intensity was measured using Gel-Pro Analyzer. β-actin was used as the loading control.

### Reverse Transcription-Quantitative Polymerase Chain Reaction (RT-qPCR)

The total RNA was extracted from the treated HTM cells using TRIzol reagent. The cDNA synthesis was performed using PrimeScript RT reagent Kit (RR047, Takara Biotechnology) according to the manufacturer’s instructions. PCR was performed with TB-Green Premix Ex Taq II in a total reaction volume of 25 μl using the following amplification steps: initial denaturation at 95°C for 30 s; 40 cycles of denaturation at 95°C for 5 s, and further elongation at 60°C for 30 s; the final extension at 95°C for 10 s. Data were processed using the 2^−ΔΔCt^ method (Livak and Schmittgen, 2001). β-actin was used as the control housekeeping gene. The primer sequences were as follows: Collagen I, 5′-GGT​TTC​AGA​GAC​AAC​TTC​CCA​A-3′ and 5′-GTC​ATT​TCC​ACA​TGC​TTT​ATT​CC-3'; α-SMA, 5′-CCG​GGA​CTA​AGA​CGG​GAA​TC-3′ and 5′-TTG​TCA​CAC​ACC​AAG​GCA​GT-3'; YAP, 5′-CCC​TCG​TTT​TGC​CAT​GAA​CC-3′ and 5′-GTT​GCT​GCT​GGT​TGG​AGT​TG-3'; TAZ, 5′-GTCACCAACAGTAGCTCAGATC-3′ and 5′-AGTGATTACAGCCAGGTTAGAAAG-3′; CTGF, 5′-AGA​ATG​ACA​GTC​CGT​CAA​AAC​AG-3′ and 5′-AGG​CCA​TTT​GTT​CAT​TAA​AAG​TG-3'; β-actin, 5′-AGC​ACA​ATG​AAG​ATC​AAG​ATC​AT-3′ and 5′-ACT​CGT​CAT​ACT​CCT​GCT​TGC-3'.

### Immunofluorescence Staining

The cells were fixed with 4% formaldehyde for 20 min, permeabilized with 0.3% Triton X-100 for 20 min, and washed thrice with phosphate-buffered saline (PBS) at room temperature. Then, the cells were blocked in PBS containing 5% bovine serum albumin for 1 h at room temperature. After blocking, the cells were incubated overnight in PBS containing primary antibodies at 4°C. The primary antibodies used were as follows: Collagen I (1:250, ab260043, Abcam), α-SMA (1:400, ab124964, Abcam), and YAP/TAZ (1:100, 8,418, CST). The cells were rinsed and incubated with secondary antibodies conjugated to Alexa Fluro 594 (1:500, 8,889, CST) in PBS for 2 h at room temperature. After washing thrice with PBS, coverslips were mounted on slides using a mounting buffer containing DAPI for 5 min, and images were obtained using a Nikon fluorescence microscope.

### Atomic Force Microscope Test

The cells in each group were inoculated into three dishes for atomic force microscope (AFM, Resolve, Bruker) test. After transfecting with lentivirus for 72 h, the cells were gently rinsed with pre-warmed PBS, immersed in medium, and placed under the AFM cantilever tip. The elastic modulus of HTM cells transfected with RhoAG14V or siYAP was obtained at room temperature in the peak-force mode. A conical probe (MLCT-bio, Bruker) constrained to the cantilever was used. Testing parameters were as follows: the half-angle of the AFM tip was 18°, and the Poisson’s ratio of the cell was considered 0.5.

### Statistical Analyses

SPSS software was used for the statistical analyses. The normal distribution of data was detected first. Two-group comparisons were performed using unpaired *t*-test. Multiple group comparisons were performed using one-way analysis of variance followed by Bonferroni’s post hoc test. If the data did not fit the normal distribution, a nonparametric test was used. The data were presented as the mean ± standard error of the mean or median with interquartile range. *p* < 0.05 was considered statistically significant.

## Results

### Bioinformatics Analyses of Gene Expression Profile

**FIGURE 1:**
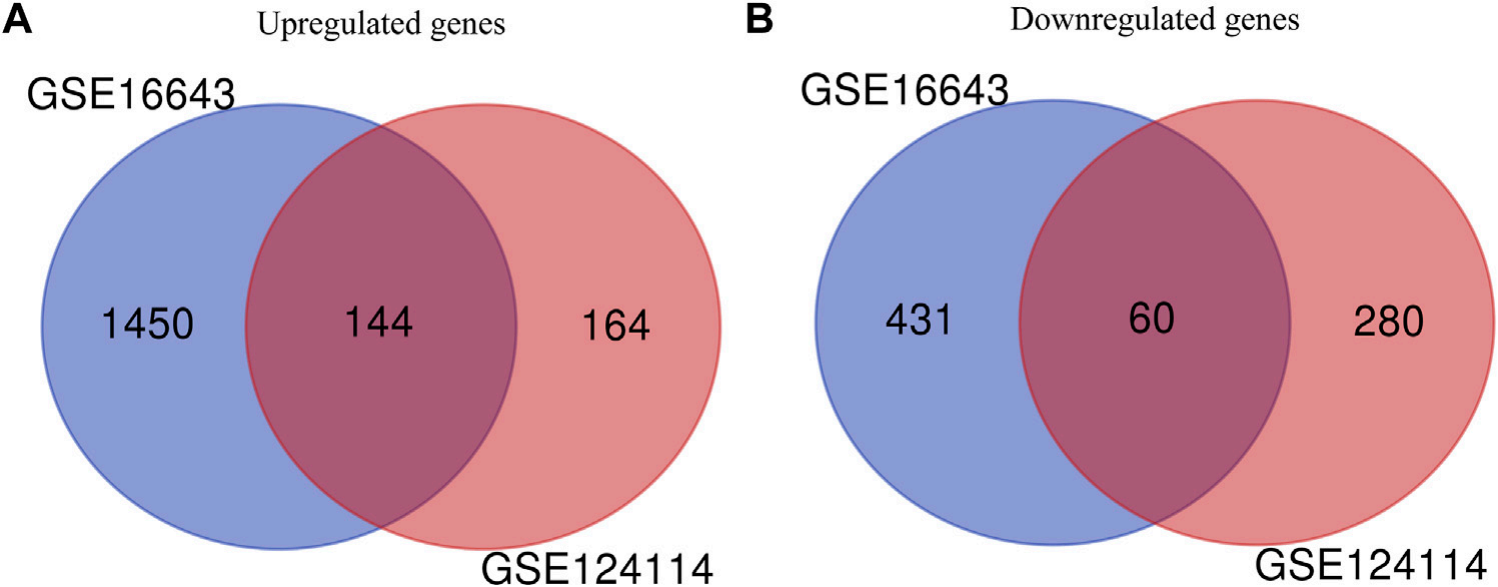
Identification of common DEGs in GSE124114 and GSE16643 **(A)** Venn plot showed the common upregulated genes in GSE124114 and GSE16643 **(B)** Venn plot showed the common downregulated genes in GSE124114 and GSE16643.

**FIGURE 2:**
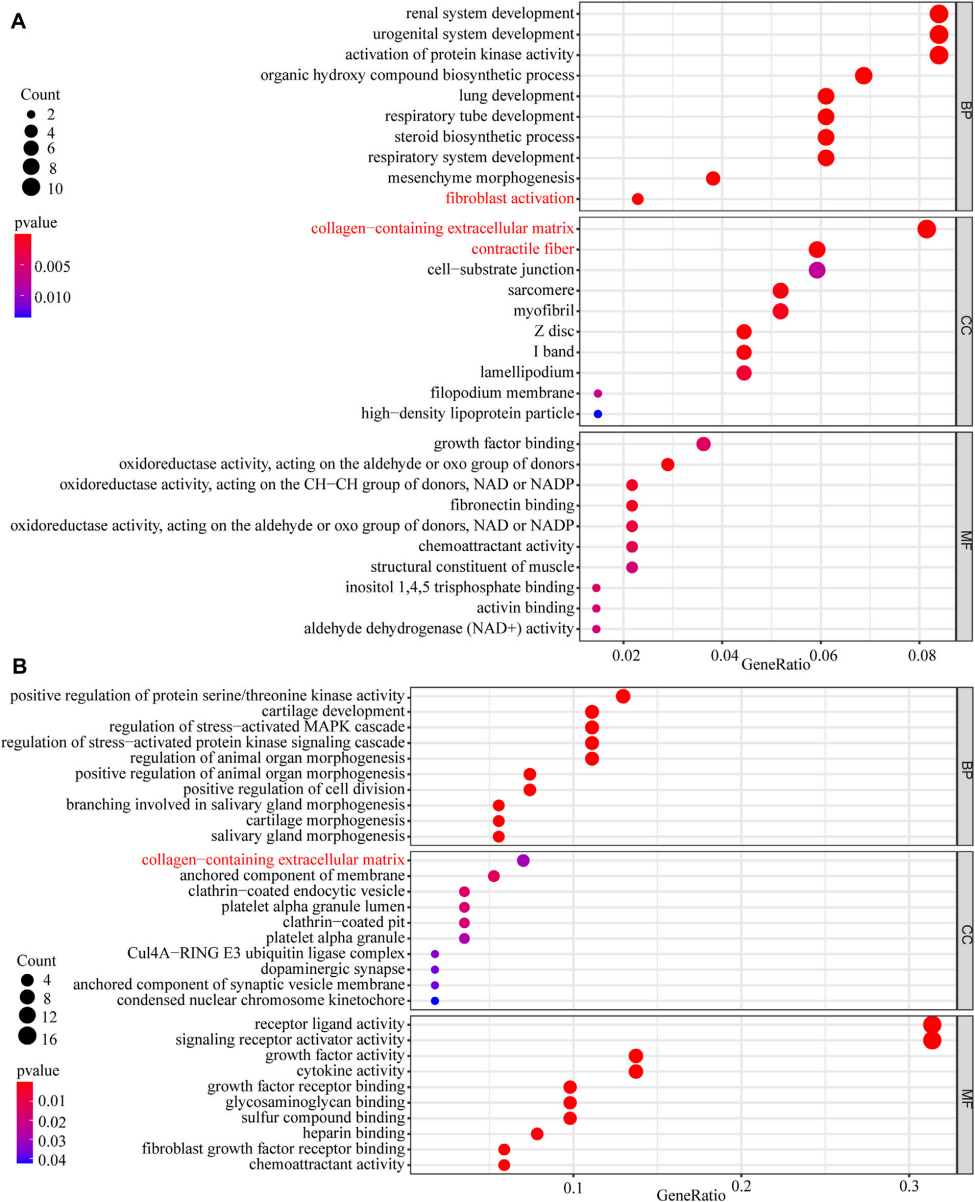
The GO enrichment analysis of DEGs **(A)** The GO enrichment analysis of upregulated DEGs **(B)** The GO enrichment analysis of downregulated DEGs.

**FIGURE 3:**
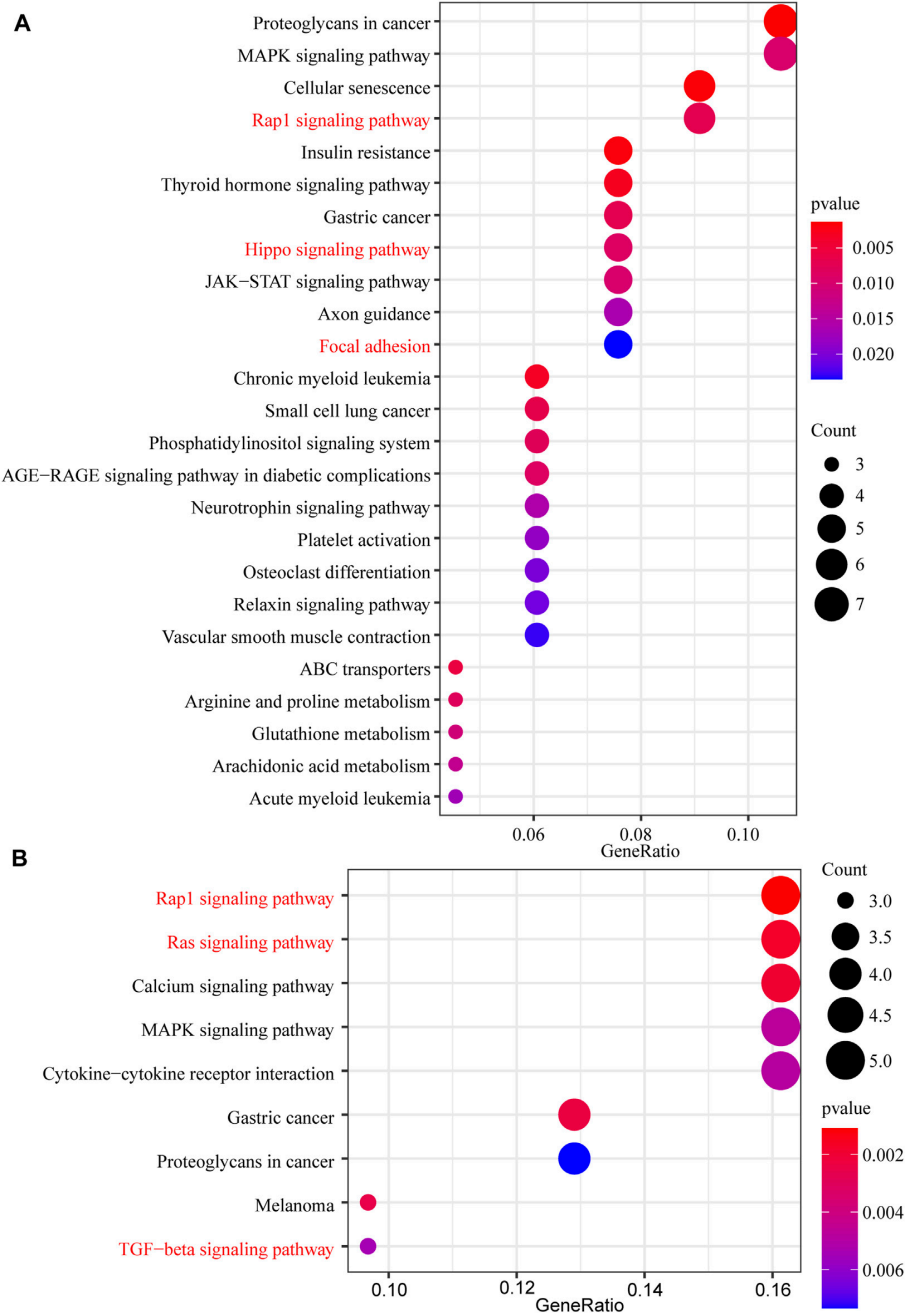
The KEGG pathway enrichment analysis of DEGs **(A)** The KEGG pathway enrichment analysis of upregulated DEGs **(B)** The KEGG pathway enrichment analysis of downregulated DEGs.

**FIGURE 4:**
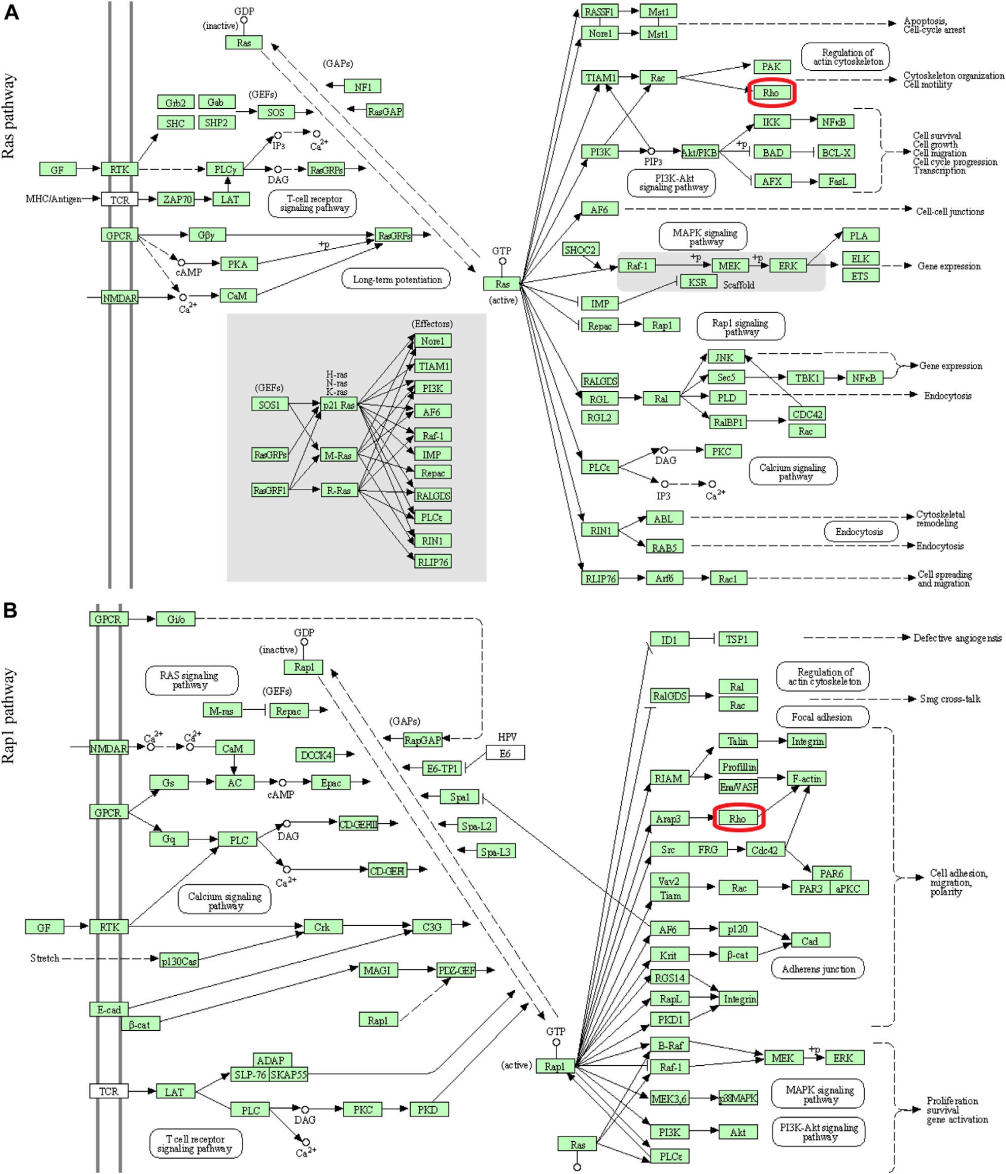
Visualization of the Ras and Rap1 pathways **(A)** The Ras pathway **(B)** The Rap1 pathway. The red box indicated that these two signaling pathways passed through Rho.

**FIGURE 5:**
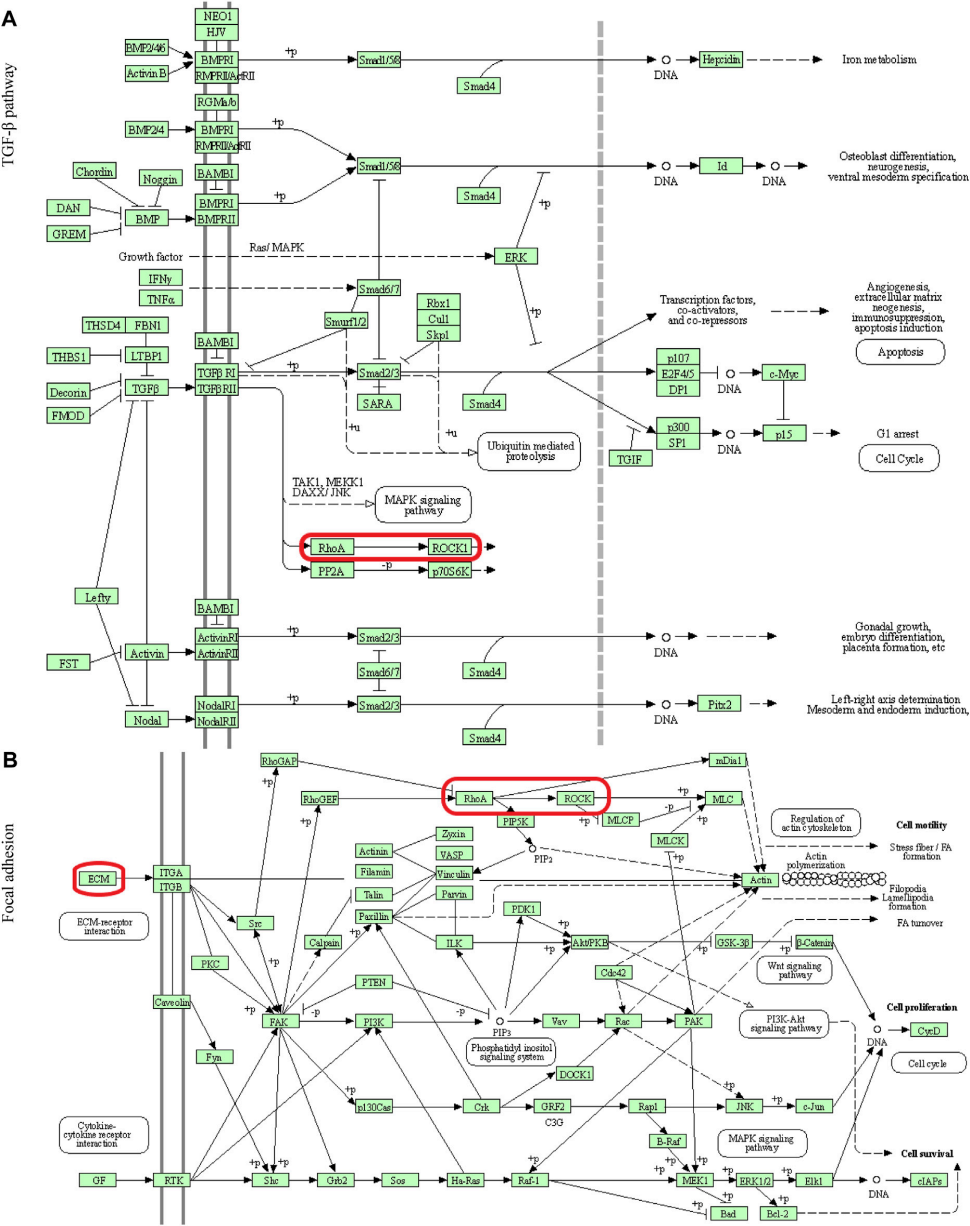
Visualization of the TGF-β pathway and Focal adhesion **(A)** The TGF-β pathway **(B)** Focal adhesion. The red box indicated that these two signaling pathways passed through RhoA/ROCK.

In this study, 648 genes (308 upregulated genes and 340 downregulated genes) from GSE124114 and 2085 genes (1,594 upregulated genes and 491 downregulated genes) from GSE16643 were differentially expressed in DEX-treated cells (Figure 1). Venn plot showed that 144 common upregulated DEGs (Figure 1A) and 60 common downregulated DEGs were identified (Figure 1B). Next, GO and KEGG functional enrichment analyses of DEGs were implemented using the R packages. The results of GO enrichment analysis suggested that DEGs were partly enriched in collagen-containing ECM and fibroblast activation (Figure 2). The KEGG analysis indicated that DEGs were partly involved in Rap1, Ras, TGF-β, and Hippo pathways, and Focal adhesion (Figure 3). To further explore the internal relationship between these pathways, we analyzed the intersection points of four pathways, including Ras pathway (Figure 4A), Rap1 pathway (Figure 4B), TGF-β pathway (Figure 5A), and Focal adhesion (Figure 5B). We found that all four pathways pass through RhoA/ROCK. These results suggested that DEX may induce the fibrotic phenotypes of HTM cells and the RhoA/ROCK pathway may be a key node in the process of the fibrotic activity.

### DEX Induced the Fibrotic Activity and Regulated the RhoA/ROCK Pathway in HTM Cells

**FIGURE 6:**
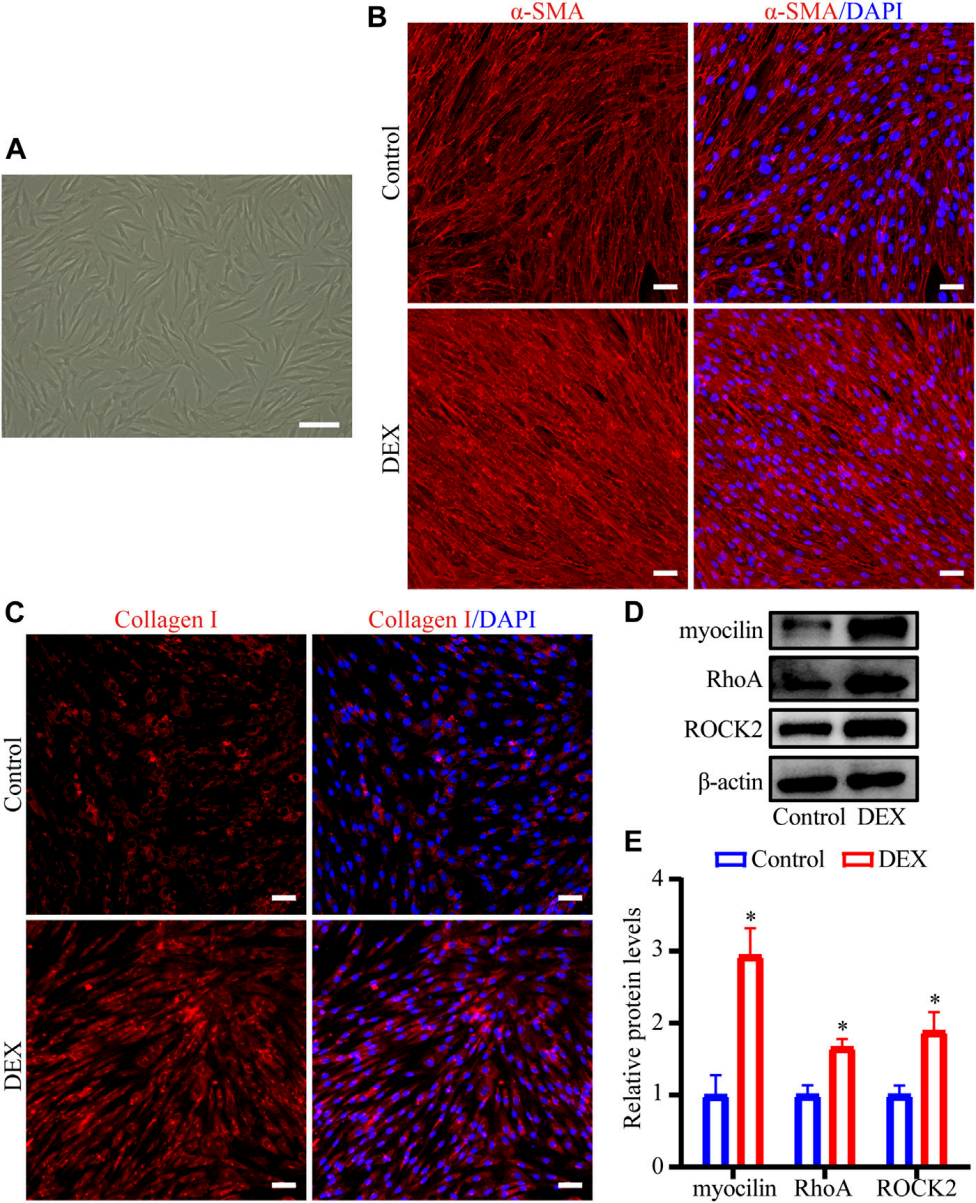
DEX upregulated Collagen I and α-SMA levels and regulated RhoA/ROCK in HTM cells **(A)** Phase-contrast microscopic image of HTM cells **(B,C)** The fluorescent images of **(B)** α-SMA and **(C)** Collagen I proteins were observed by immunofluorescence staining. Scale bar, 100 μm **(D)** The bands of myocilin, RhoA, and ROCK2 proteins and **(E)** quantification analyses of myocilin, RhoA, and ROCK2 expression levels. ^*^ *p* < 0.05.

First, we characterized HTM cells as previously described (Stamer and Clark, 2017; Keller et al., 2018). The morphology of the cells was shown in Figure 6A. DEX treatment induced the expression of myocilin in HTM cells (*p* < 0.05, Figures 6D, E). To evaluate the effect of DEX on the fibrotic activity of HTM cells, the expression and subcellular location of Collagen I and α-SMA proteins were observed using immunofluorescence staining. Expression levels of α-SMA (Figure 6B) and Collagen I (Figure 6C) were significantly upregulated in the DEX-treated group. To identify the critical genes of the RhoA/ROCK pathway, western blot was performed in HTM cells treated with DEX. Western blot results demonstrated that the expression levels of RhoA (1.7-fold, *p* < 0.05) and ROCK2 (1.9-fold, *p* < 0.05) in the DEX-treated group were upregulated compared with those in the control group (Figures 6D, E). Through experimental validation, we demonstrated that DEX promoted ECM accumulation and activated the RhoA/ROCK pathway.

### RhoAG14V Promoted the Fibrotic Activity and Regulated YAP/TAZ in HTM Cells

**FIGURE 7:**
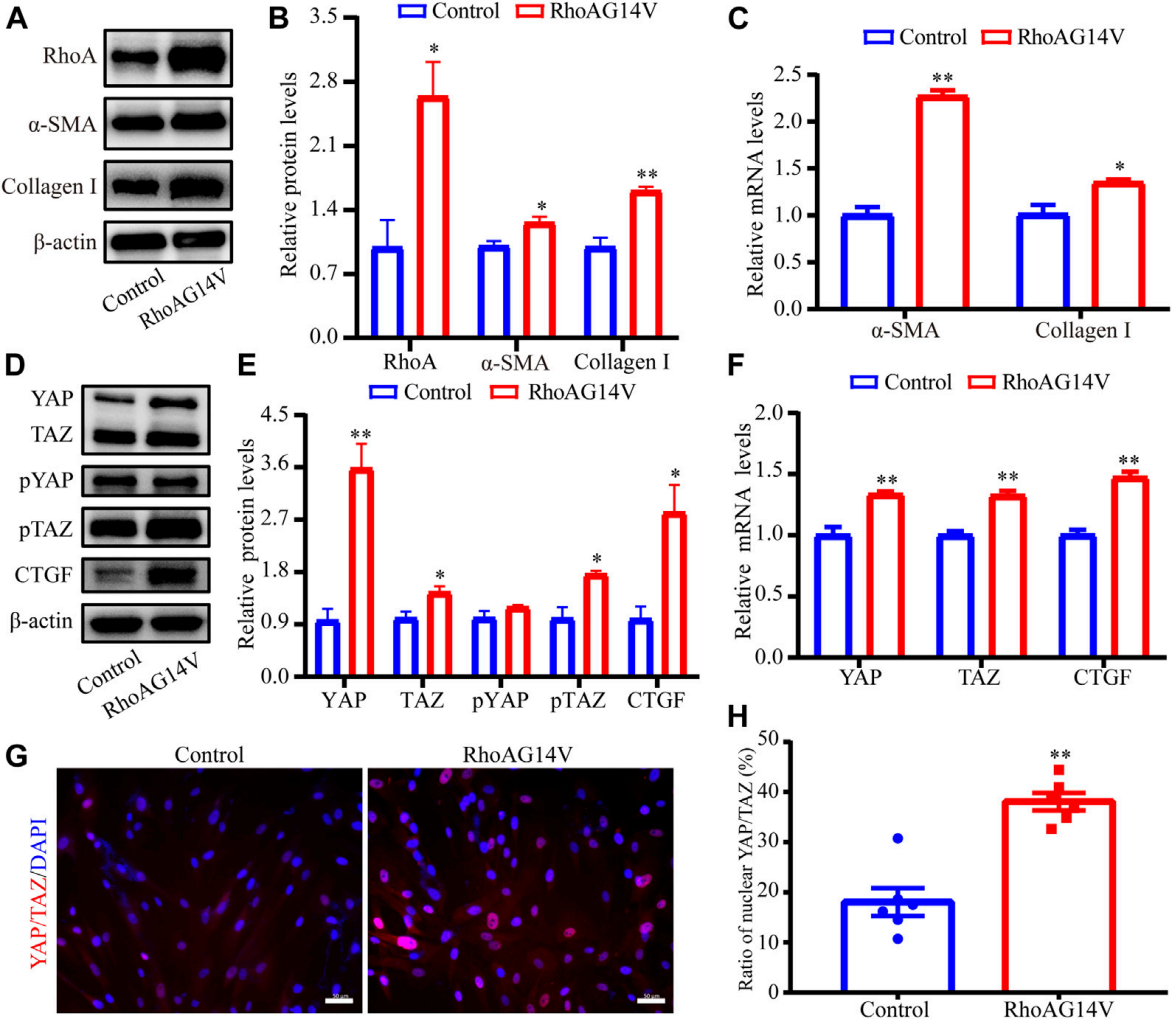
RhoAG14V promoted the expression of Collagen I and α-SMA and regulated YAP/TAZ in HTM cells **(A)** The bands of RhoA, α-SMA, and Collagen I proteins **(B)** Quantification analyses of RhoA, α-SMA, and Collagen I expression levels **(C)** RT-qPCR analyses of α-SMA and Collagen I mRNAs **(D)** The bands of YAP/TAZ, pYAP, pTAZ, and CTGF proteins **(E)** Quantification analyses of YAP, TAZ, pYAP, pTAZ, and CTGF proteins **(F)** RT-qPCR analyses of YAP, TAZ, and CTGF mRNAs **(G)** Representative fluorescent graphs of YAP/TAZ labeled by specific antibody and **(H)** quantitative analysis of YAP/TAZ in the nucleus. Scale bar, 50 μm; ^*^ *p* < 0.05; **p < 0.01.

First, the lentivirus transfection efficiency of RhoA was evaluated using western blot (2.7-fold, *p* < 0.05, Figures 7A,B). To evaluate the effect of RhoAG14V on the α-SMA and Collagen I levels in HTM cells, western blot and RT-qPCR were performed. The expression levels of α-SMA (1.3-fold, *p* < 0.05) and Collagen I (1.6-fold, *p* < 0.01) proteins in the RhoAG14V group were significantly increased (Figures 7A,B). In addition, Figure 7C showed that RhoAG14V upregulated the mRNA levels of α-SMA (2.3-fold, *p* < 0.01) and Collagen I (1.3-fold, *p* < 0.05).

To explore the correlation between RhoA/ROCK and YAP/TAZ, the protein and mRNA levels of YAP, TAZ, and CTGF were quantified using western blot and RT-qPCR, and the intracellular location of YAP/TAZ was visualized using the immunofluorescence staining. The bands of YAP/TAZ, pYAP, pTAZ, and CTGF proteins in response to RhoAG14V, as determined using western blot were presented in Figure 7D. Quantification results of western blot revealed that RhoAG14V enhanced the expression of YAP (3.6-fold, *p* < 0.01), TAZ (1.4- fold, *p* < 0.05), pTAZ (1.8-fold, *p* < 0.05), and CTGF (2.8-fold, *p* < 0.05), but the expression of pYAP had no obvious change (Figure 7E). In addition, the mRNA levels of YAP (1.3-fold, *p* < 0.01), TAZ (1.3-fold, *p* < 0.01), and CTGF (1.5-fold, *p* < 0.01) were upregulated by RhoAG14V (Figure 7F). Immunofluorescence staining indicated that the RhoAG14V-transfected cells exhibited mainly nuclear YAP/TAZ (2.1-fold, *p* < 0.01), whereas the control cells retained YAP/TAZ in the cytoplasm (Figures 7G,H). Collectively, YAP/TAZ may be the downstream effectors of RhoA participating in the fibrotic activity.

### YAP Was Required for DEX- and RhoAG14V-Mediated Fibrotic Activity of HTM Cells

**FIGURE 8:**
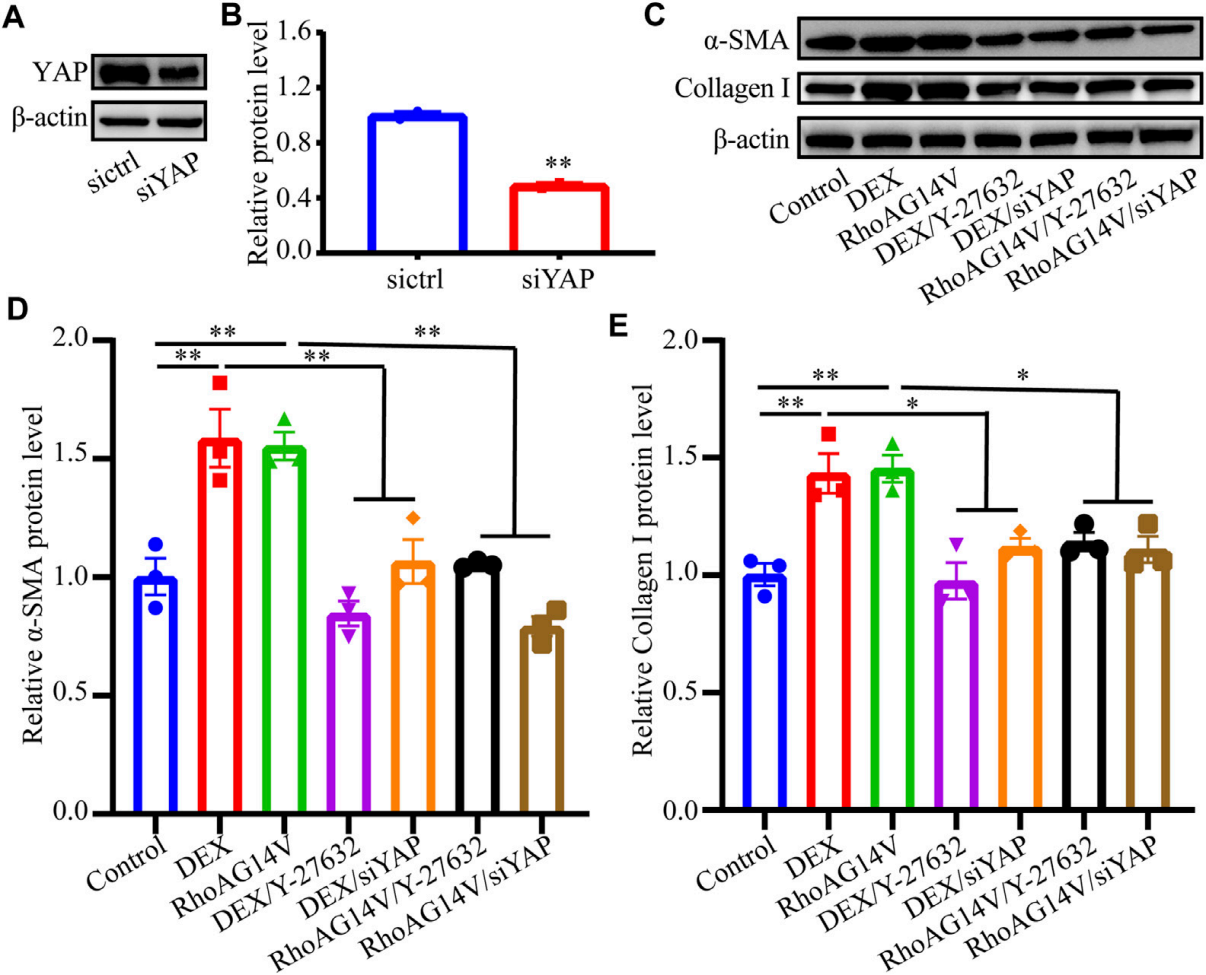
Inhibition of ROCK or YAP decreased the expression of Collagen I and α-SMA proteins induced by DEX or RhoAG14V in HTM cells **(A)** The band of YAP protein **(B)** The expression level of YAP was quantified by western blot using the indicated antibodies **(C)** The bands of α-SMA and Collagen I proteins **(D,E)** Quantification analyses of **(D)** α-SMA and **(E)** Collagen I proteins in HTM cells treated with or without DEX, ROCK inhibitor Y-27632, RhoAG14V and siYAP were evaluated by western blot. ^*^ *p* < 0.05; **p < 0.01.

**FIGURE 9:**
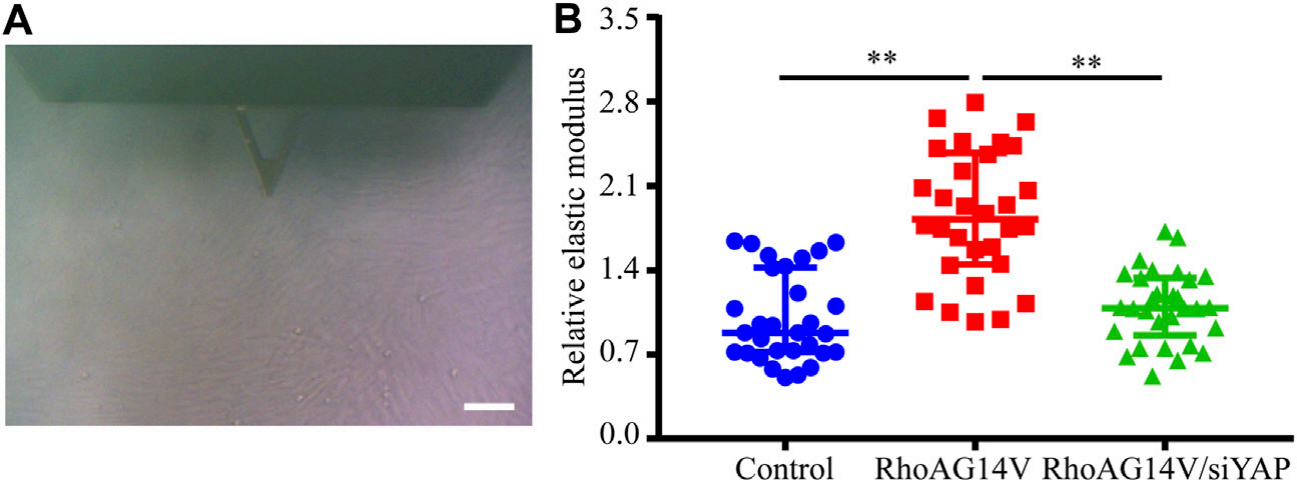
siYAP decreased the elastic modulus of HTM cells caused by RhoAG14V (A) Representative image of indentation experiment that represented the AFM probe on the HTM cells captured by the inverted microscope. Black triangle, the cantilever of AFM; Scale bar, 100 μm **(B)** The elastic modulus of HTM cells transfected with or without RhoAG14V and siYAP. **p < 0.01.

To investigate whether a positive feedback loop exists between the DEX, RhoA, and YAP axis, the expression of YAP was knocked down. Western blot results indicated the efficiently decreased expression of YAP (Figures 8A,B). To confirm whether fibrotic activity caused by DEX or RhoAG14V is inhibited in HTM cells transfected by siYAP, the expression of α-SMA and Collagen I and was detected. The bands of western blot were shown in Figure 8C. In the absence of siYAP or Y-27632, DEX or RhoAG14V significantly increased the expression of α-SMA and Collagen I proteins compared with the control group. However, in the presence of siYAP or Y-27632, α-SMA and Collagen I proteins were reduced compared with DEX-treated or RhoAG14V-transfected group (Figures 8D, E). Moreover, the elastic modulus of HTM cells transfected with RhoAG14V or siYAP was probed using AFM. Figure 9A was the image of the indentation experiment that represented the AFM probe on the HTM cells captured by the inverted microscope. The cellular elastic modulus presented a significant increase in the RhoAG14V group. However, siYAP decreased the cellular elastic modulus caused by RhoAG14V (*p* < 0.01, Figure 9B). These results indicated that the RhoA/ROCK-YAP axis played an important role in the fibrotic activity of DEX-treated HTM cells.

## Discussion

The TM tissue is responsible for the generation of outflow resistance through the homeostatic turnover of ECM (Keller et al., 2009; Vranka et al., 2015). ECM accumulation, fibroblast activation, and increased stiffness, which can increase resistance to the AH outflow thereby leading to elevation in IOP (Pattabiraman and Rao, 2010; Last et al., 2011), are typical characteristics of fibrotic activity. Notably, collagen is the major protein found within the ECM. Aberrant collagen turnover of the TM tissue resulted in reduced AH drainage and elevated IOP in MMP-9 null mice (De Groef et al., 2016). Moreover, TM tissue has been reported to express α-SMA, which is a biomarker of fibroblast activation (Flügel et al., 1991; de Kater et al., 1992; Stamer and Clark, 2017). In addition, DEX has been reported to promote the expression of α-SMA leading to stiffening of the TM cells and matrix (Raghunathan et al., 2015). In this study, we applied a bioinformatics method to explore pathological processes based on differential gene expression profiles between normal control and DEX-treated cell samples. The results of GO enrichment analysis revealed that abnormal fibrotic activity (collagen-containing ECM, fibroblast activation, contractile fiber) was significant in DEX-treated HTM cells. Experimental results confirmed that DEX promoted the expression of Collagen I and α-SMA proteins, which was consistent with the results of GO enrichment analysis. These findings suggest that aberrant ECM accumulation is involved in the pathological processes of glaucoma, and DEX is an important factor that induces fibrotic activity in HTM cells.

ECM remodeling is widely involved in the progression of tissue fibrosis. Wnt/β-catenin, TGF-β, and MAPK pathways have been reported to participate in ECM remodeling (Pattabiraman and Rao, 2010; Raghunathan et al*.*, 2015; Pattabiraman et al., 2014; Villarreal et al., 2014; Hernandez et al., 2018). The KEGG analysis provided an approach to explore the internal associations between DEGs. It was determined that these DEGs were enriched in Rap1, Ras, and TGF-β pathways, and Focal adhesion. These pathways are related to the RhoA/ROCK pathway. In a previous study, DEX was shown to induce transient activation of RhoA in HTM cells (Fujimoto et al., 2012), which further activated the downstream effector ROCK. RhoAG14V, a constitutively active variant of RhoA, has been reported to induce ECM synthesis (Pattabiraman and Rao, 2010) and decrease AH outflow through the TM in anterior chambers (Pattabiraman et al., 2015). Other studies have suggested that the RhoA/ROCK plays an important role in regulating IOP, which was reduced in the presence of RhoA inhibitor C3 transferase (Tan et al., 2018; Tan et al., 2019; Tan et al., 2020) and ROCK inhibitor Y-27632 (Pattabiraman et al., 2015; Chen et al., 2020). Based on these findings, it was demonstrated that DEX not only increased the expression level of RhoA but also activated ROCK. In addition, RhoA induced ECM accumulation and activated myofibroblast-like cells. These findings indicated that DEX may induce the fibrotic activity in HTM cells through the RhoA/ROCK pathway and that RhoA probably serves as an intervening factor for lowering IOP (Rao et al., 2017; Tan et al., 2018; Tan et al., 2019; Chen et al., 2020; Tan et al., 2020).

Mechanotransduction enables cells to sense and adapt to external forces and physical constraints (Vogel and Sheetz, 2006). The mechanical signals include endogenous and exogenous stimuli. Endogenous stimuli are mainly derived from ECM stiffness and cytoskeletal tension, whereas some mechanical interventions such as cyclic stretch, stiffer substrates, and topography provide the exogenous stimuli (Raghunathan et al., 2013; Thomasy et al., 2013; Raghunathan et al., 2014; Cui et al., 2015). These mechanoresponses involve not only the rapid remodeling of ECM and cytoskeleton but also the activation of specific genetic programs. Earlier studies have reported that mechanical stimulation can regulate the Hippo pathway, in which YAP and TAZ function as essential effectors of mechanotransduction to regulate cell proliferation and differentiation (Wada et al., 2011; Codelia et al., 2014). The KEGG analysis revealed that a part of DEGs was enriched in the Hippo pathway. However, the intracellular relationship between RhoA activation and YAP/TAZ is not well elucidated in HTM cells. Understanding the mechanism of RhoAG14V-induced fibrotic activity is particularly meaningful for the identification of intervention targets. In this study, it was revealed that RhoAG14V promoted the expression of YAP/TAZ (suggesting YAP/TAZ activation), but *p*-TAZ was also upregulated. Therefore, we performed immunocytochemistry to confirm the subcellular localization of YAP/TAZ according to other studies (Raghunathan et al., 2014; Ho et al., 2018; Yemanyi and Raghunathan, 2020). The nuclear translocation of YAP/TAZ was significantly increased by RhoAG14V. Moreover, the expression of CTGF was also upregulated that further indicated the activation of YAP/TAZ. In the AH of patients, the level of CTGF exhibits a significant increase (Ho et al., 2020). In turn, the secretion of CTGF further stimulates ECM accumulation (Wallace et al., 2013).

**FIGURE 10:**
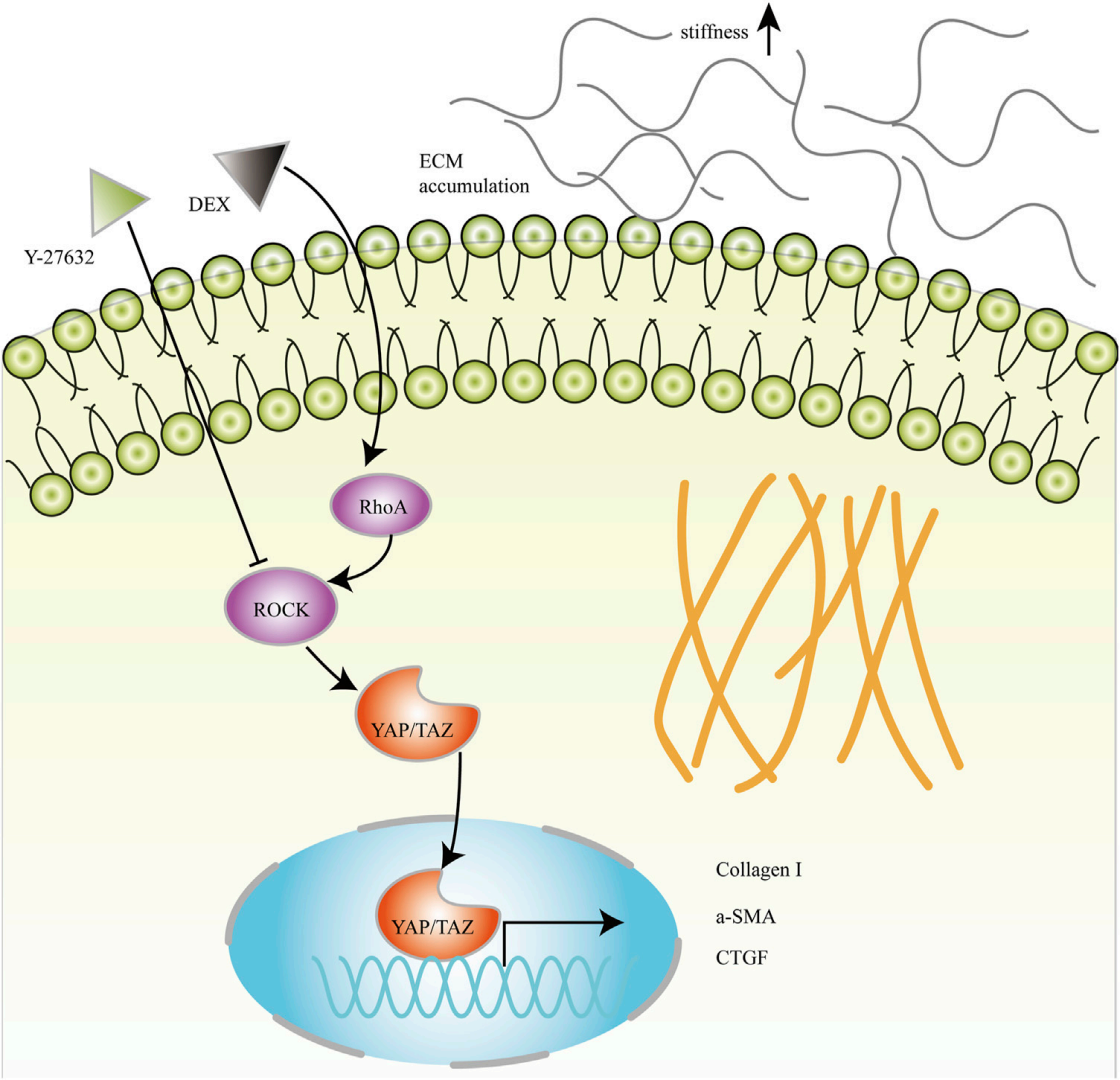
Schematic diagram of RhoA/ROCK and YAP/TAZ mediated fibrotic activity in DEX-treated HTM cells. RhoA/ROCK activated by DEX induces nuclear translocation of YAP/TAZ, which leads to an increase of Collagen I, myofibroblast biomarker α-SMA, and CTGF. Aberrant ECM accumulation and remodeling may increase the elastic modulus of HTM cells.

Lysophosphatidic acid (LPA) generated by autotaxin (ATX) is elevated in the AH of glaucoma (Iyer et al., 2012; Honjo et al., 2018a; Honjo et al., 2018b; Ho et al., 2020; Igarashi et al., 2020). ATX is robustly induced by DEX treatment that is associated with fibrotic changes and ECM accumulation in HTM cells (Honjo et al*.*, 2018b). The previous study has demonstrated that DEX can regulate the changes of the cytoskeleton by increasing the expression of YAP/TAZ (Peng et al., 2018). Additionally, LPA stimulates the activation of YAP/TAZ and induces the expression of CTGF and ECM genes in HTM cells (Ho et al., 2018; Yemanyi and Raghunathan, 2020). These results suggested that ATX/LPA-YAP/TAZ axis played a key role in the fibrotic activity of HTM cells. In this study, RhoA/ROCK is an important modulator of DEX-induced fibrotic changes in HTM cells. RhoA/ROCK activation may contribute to cytoskeletal tension, thus induces nuclear translocation of Hippo pathway effectors YAP/TAZ, which leads to an increase of Collagen I, myofibroblast biomarker α-SMA, and CTGF. Aberrant ECM accumulation and remodeling results in increased cellular stiffness, which may stimulate RhoA/ROCK and YAP/TAZ activation. Eventually, a vicious circle forms between DEX-induced ECM remodeling and RhoA/ROCK-YAP/TAZ activation (Figure 10). However, the association between ATX/LPA and RhoA/ROCK and the effectiveness of YAP/TAZ in regulating IOP *in vivo* need further exploration.

In summary, the present study offered systematic and comprehensive bioinformatics analyses between normal and DEX-treated HTM cells. The results suggested that DEGs were associated with multiple pathways which may be involved in the occurrence and development of fibrotic activity in HTM cells. In addition, it was further demonstrated that DEX induced the fibrotic activity of HTM cells through the RhoA/ROCK-YAP/TAZ axis. These findings not only improve our understanding of the pathological processes of fibrotic activity in HTM cells but also provide potential therapeutic targets for glaucoma.

## Data Availability

The datasets analyzed for this study can be found in The Gene Expression Omnibus (https://www.ncbi.nlm.nih.gov/geo/).
